# Relationship between the Cervical Microbiome, HIV Status, and Precancerous Lesions

**DOI:** 10.1128/mBio.02785-18

**Published:** 2019-02-19

**Authors:** Cameron Klein, Daniela Gonzalez, Kandali Samwel, Crispin Kahesa, Julius Mwaiselage, Nirosh Aluthge, Samodha Fernando, John T. West, Charles Wood, Peter C. Angeletti

**Affiliations:** a Nebraska Center for Virology, School of Biological Sciences, University of Nebraska-Lincoln, Lincoln, Nebraska, USA; b Ocean Road Cancer Institute, Dar es Salaam, Tanzania; c Animal Sciences, University of Nebraska-Lincoln, Lincoln, Nebraska, USA; The Pennsylvania State University College of Medicine; Washington University School of Medicine

**Keywords:** 16S RNA, cervical cancer, deep sequencing, human papillomavirus, human immunodeficiency virus, microbiome

## Abstract

Nearly all cervical cancers are causally associated with human papillomavirus (HPV). The burden of HPV-associated dysplasias in sub-Saharan Africa is influenced by HIV. To investigate the role of the bacterial microbiome in cervical dysplasia, cytobrush samples were collected directly from cervical lesions of 144 Tanzanian women. The V4 hypervariable region of the 16S rRNA gene was amplified and deep sequenced. Alpha diversity metrics (Chao1, PD whole tree, and operational taxonomic unit [OTU] estimates) displayed significantly higher bacterial richness in HIV-positive patients (*P* = 0.01) than in HIV-negative patients. In HIV-positive patients, there was higher bacterial richness in patients with high-grade squamous intraepithelial lesions (HSIL) (*P* = 0.13) than those without lesions. The most abundant OTUs associated with high-grade squamous intraepithelial lesions were *Mycoplasmatales*, *Pseudomonadales*, and *Staphylococcus*. We suggest that a chronic mycoplasma infection of the cervix may contribute to HPV-dependent dysplasia by sustained inflammatory signals.

## Abstract (precis)

HPV is known to be the causal agent in the majority of cervical cancers. However, the role of the cervical bacterial microbiome in cervical cancer is not clear. To investigate that possibility, we collected cervical cytobrush samples from 144 Tanzanian women and performed deep sequencing of bacterial 16S rRNA genes. We found that HIV-positive patients had greater bacterial richness (*P* = 0.01) than HIV-negative patients. We also observed that women with high-grade squamous intraepithelial lesions (HSIL) had greater cervical bacterial diversity than women with cytologically normal cervices. Data from our precise sampling of cervical lesions leads us to propose that *Mycoplasma* contributes to a cervical microbiome status that promotes HPV-related cervical lesions. These results suggest a greater influence of the bacterial microbiota on the outcome of HPV infection than previously thought.

## INTRODUCTION

Human papillomavirus (HPV) is the causative agent responsible for 99% of cervical cancers (1). HPV contributes to about 4.8% of all cancers (1). The disease burden of HPV is most dramatic in developing regions of the world, with HPV contributing to 14.2% of cancers in sub-Saharan Africa (1). Cervical cancer disproportionately affects sub-Saharan Africa, where 9% of the world’s female population over 15 years old accounts for 14% of the world’s incidence of cervical cancer and 18% of cervical cancer-related deaths (2). The current study uses cervical swab samples obtained from Tanzania, which has among the highest cervical cancer mortality rates by country.

Sub-Saharan Africa also has among the highest HIV rates in the world. The association between HIV and cervical cancer has been better studied than any other factor associated with HPV-related cancers. HIV infection has been strongly linked to increased risk of infection with HPV and the severity of HPV pathogenesis (3–5). High-risk HPV genotypes are more prevalent in HIV-positive (HIV+) women, suggesting that HIV infection provides an environment where these high-risk HPVs can better establish infection and replicate (6). A likely factor in this is a decrease in T-cell surveillance, which results in an increase in HPV replication with decreasing CD4^+^ cell count, and other changes in the cervical immune microenvironment as HIV infection progresses. Multiple studies have shown an increase in HPV detection in cervical intraepithelial neoplasms in individuals with less than 200 CD4^+^ cells per μl of serum (7–10). Thus, the cervical immune microenvironment may be a cofactor in suppression of cervical cancer.

Changes in the cervicovaginal bacterial microbiome have been suggested to contribute to the development of precancerous cervical lesions (11–17). Chronic inflammation of the cervix (cervicitis), which is a result of cervicovaginal pathogens, leads to conditions like pelvic inflammatory disease (PID) and bacterial vaginosis (BV), both of which are associated with persistent HPV infection and cervical cancer (18, 19). Both PID and BV are more prevalent in sub-Saharan Africa and in HIV-positive populations (20–22). Comparative genomic analyses in women infected with HIV have shown that a shift in microbial diversity as a result of BV is detectable; whether this shift directly affects formation of precancerous cervical lesions is not clear (23). Given that cervical cancer rates are expected to rise in sub-Saharan Africa as the HIV-positive population receives life-extending antiretroviral therapy (ART), it is even more important to understand the risk factors associated with the cervical microbiome. There are previous studies that have analyzed how cervical microbiota differ at different stages of cervical cytology or as a function of HIV status (24–28). The current study defines bacterial communities associated with cervical lesions and with HIV, which represents a significant advance. Cervical cytology is graded by pap smear screening for nuclear abnormalities according to the Bethesda guidelines.

In this study, we utilized 16S rRNA gene deep sequencing on a set of 144 cervical swab samples from a cohort of Tanzanian women to gain an understanding of the differences in the cervical bacterial community composition as a function of cervical cytology grade and HIV status. The data presented here identify bacterial taxonomies associated with high-grade cervical lesions. In these studies, cervical lesions were sampled directly by cytobrush, instead of cervicovaginal lavage sampling. The rationale behind this approach was that the sites of the lesions are where tumors form, thus bacteria associated with lesion sites are more likely to be relevant to the process of disease progression than those associated with other regions.

## RESULTS

### Demographics.

**FIG 1:**
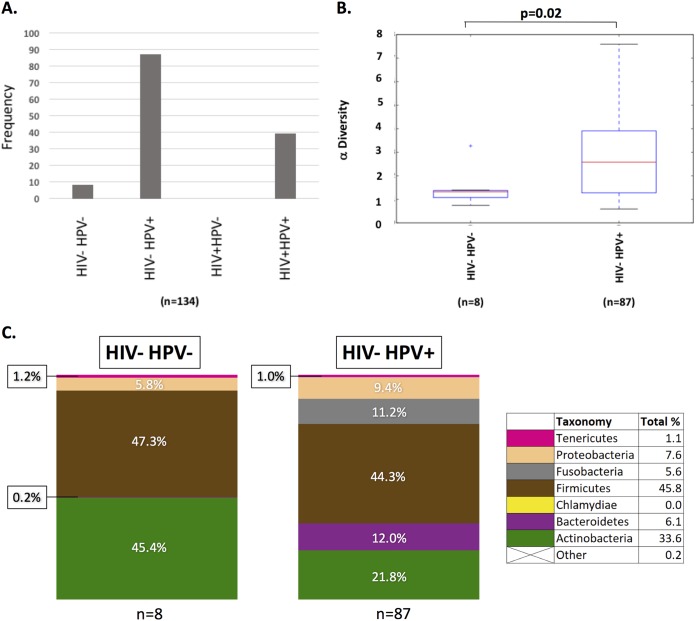
Effect of HPV status upon the cervical microbiome diversity. (A) A total of 134 cohort DNA samples were genotyped for HIV and HPV status. The frequency of samples were graphed as the following groups: HIV− HPV− (*n* = 8), HIV− HPV+ (*n* = 87), HIV+ HPV− (*n* = 0), and HIV+ HPV+ (*n* = 39). Taxonomic groups were determined by analysis of 16S deep sequencing results of bacterial DNAs. (B) Alpha diversity is graphed as a function of HIV− HPV− (*n* = 8) and HIV− HPV+ (*n* = 87). A *t* test showed a significant difference between the HPV− and HPV+ groups (*P* = 0.02). (C) Bacterial diversity is graphed with each phylum represented as a different color. The color code representing each bacterial phylum is shown in the legend to the right.

Of the 144 patient samples, 41 were HIV positive (HIV+) and 103 were HIV negative (HIV−), with an average patient age of 37 years old. Of these 144 samples, 134 had HPV tests and deep sequencing reads of >1,000. The frequencies of HPV+ and HPV− samples with respect to HIV status are plotted in Fig. 1A. There were 8 HIV− HPV− samples and 87 HIV− HPV+ samples, but there were no HIV+ HPV− samples and 39 HIV+ HPV+ samples. Among HIV− samples, HPV had a statistically significant effect (*P* = 0.02) on the cervical microbiome (Fig. 1B and C). Those microbes which were enriched in HPV+ samples were *Bacteriodetes* and fusobacteria. Also, there was a decrease in *Actinobacteria*. Cervical cytology was determined to be negative for intraepithelial lesion or malignancy (NILM) in 23 samples, low-grade squamous intraepithelial lesions (LSIL) in 72 samples, and high-grade squamous intraepithelial lesions (HSIL) in 50 samples. Visual inspection with acetic acid (VIA), the standard for cervical lesion detection in Tanzania, was carried out immediately following sample collection. Twenty-six patients were found to be VIA positive for cervical lesions and 115 were VIA negative. All VIA-positive samples were identified as LSIL or HSIL, while several VIA-negative samples were found to be NILM, LSIL, or HSIL by pap smear. Odds ratios were used to identify risk factors for testing VIA positive. Testing HIV+, HSIL, having >5 sexual partners, and having been infected with a sexually transmitted infection (STI) were identified as significant risk factors for positive VIA status (*P* = 0.0001, *P* = 0.038, *P* = 0.006, and *P* = 0.0008, respectively).

### Cervical bacteria composition and richness.

Samples rarefied to an even depth (1,000 reads) were used to generate 813 operational taxonomic units (OTUs). To assess whether the sampling depth was adequate, rarefaction curves were generated using observed OTUs for HIV status and cervical cytology (see Fig. S1 in the supplemental material). Rarefaction curves for both did not converge but showed a diminishing rate of new OTU identification as the number of reads per sample increased, implying that sampling depth was adequate for evaluating dominant members of the cervical bacterial community. Good’s coverage test showed that the sequencing depth was able to characterize 99.4% of the bacterial community on average.

10.1128/mBio.02785-18.1 FIG S1 Bacterial 16S deep sequencing data were analyzed with rarefaction curves generated from the OTU data. These rarefactions were then compared with HIV status and cervical cytology. (S1A) The red squares and “0”” represent HIV-negative samples. The black squares and “1” represent HIV-positive samples. The line indicated as “NA” is the unadjusted control. (S1B) Red squares represent HSIL, and blue squares represents LSIL. Green squares represent NILM. The line indicated as “NA” is the unadjusted control. Download FIG S1, TIF file, 1.1 MB. Copyright © 2019 Klein et al. 2019 Klein et al. This content is distributed under the terms of the Creative Commons Attribution 4.0 International license.

**FIG 2:**
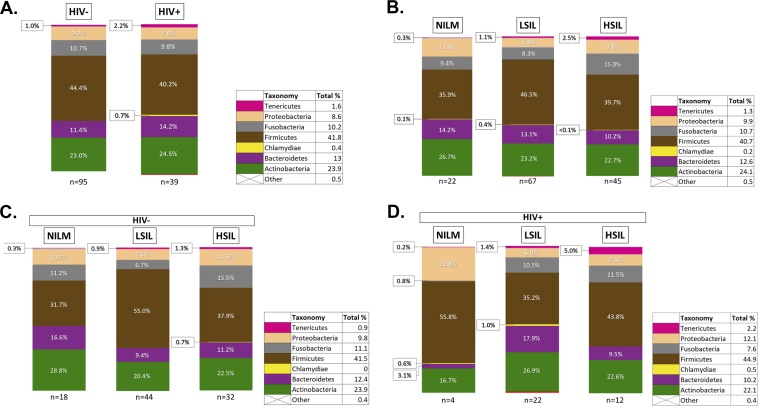
Phylum-level taxonomy of the cervical bacterial community composition as a function of HIV status and cervical cytology. (A) Phylum-level bacterial taxonomy of the cohort is displayed by HIV status. (B) Phylum-level bacterial taxonomy of the cohort is displayed as a function of cervical cytology. (C) Phylum-level taxonomy of HIV-negative patients as a function of cervical cytology grade. (D) Phylum-level taxonomy of HIV-positive patients as a function of cervical cytology grade. Each phylum is represented as a percentage of the total.

**FIG 3:**
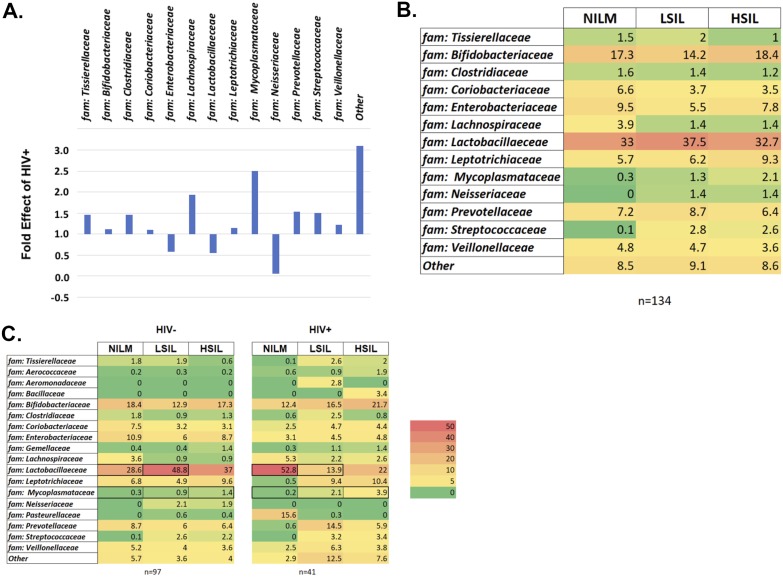
Relative abundance heatmap of family-level taxonomy of the cervical bacterial community composition as a function of HIV status and cervical cytology. (A) Fold effect of HIV+ on the family-level bacterial taxonomy within the cohort (normalized to 1). (B) Relative abundance heatmap of the family-level taxonomy of cohort versus cervical cytology. (C) Relative abundance heatmap of the family-level taxonomy of the cohort by cervical cytology, separated by HIV status. The data are presented as percentages of the total. The scale is shown to the right of the heatmap.

The taxonomic analysis of the reads revealed the presence of six main phyla (relative abundance of >1%) in the cervical epithelium, regardless of HIV or cervical cytology status (Fig. 2). *Firmicutes* was the predominant phylum across all sampling groups, accounting for 41.3% of total reads. The average relative abundance of *Firmicutes* decreased slightly in HIV+ samples compared to HIV− samples (44.4% to 40.2%) and varied by cervical cytology, though no obvious trend was apparent. When considering only the HIV+ samples, the relative abundance of *Firmicutes* appeared to decrease in patients with cervical lesions. *Firmicutes* reads were primarily from the genus *Lactobacillus*, which accounted for 21.9% of total reads. *Tenericutes* accounted for 1.5% of total reads and showed a clear increase in relative abundance with increasing severity of cervical lesions. In HIV− patients, *Tenericutes* increased from 0.3% of reads in NILM patients to 1.3% in HSIL patients (Fig. 2C). In HIV+ patients, the shift is larger; the relative abundance of *Tenericutes* increased from 0.2% in NILM patients to 5.0% in HSIL patients (Fig. 2D). *Tenericutes* reads were primarily assigned to the *Mycoplasma* and *Ureaplasma* genera, which account for 1.1% and 0.2% of total reads, respectively. *Proteobacteria*, fusobacteria, *Bacteroidetes*, and *Actinobacteria* had smaller or less consistent shifts in relative abundance between HIV and cervical cytology categories. The relative abundance of *Tenericutes* and *Bacteroidetes* were significantly different between HIV+ and HIV− groups (*P* = 0.020 and *P* = 0.017, respectively). No other phyla reached significance on the basis of HIV status or cervical cytology. Comparison of the relative abundance of bacterial families (Fig. 3) found that *Mycoplasmataceae* and *Prevotellaceae* were significantly more abundant in HIV+ patients (*P* = 0.03 and *P* = 0.07, respectively). No families were found to be significantly different in abundance on the basis of cervical cytology alone. However, when analyzed among HIV+ patients, *Prevotellaceae* was found to be significantly more abundant in cervical lesions (*P* = 0.068).

### Cervical bacterial diversity estimates.

**FIG 4:**
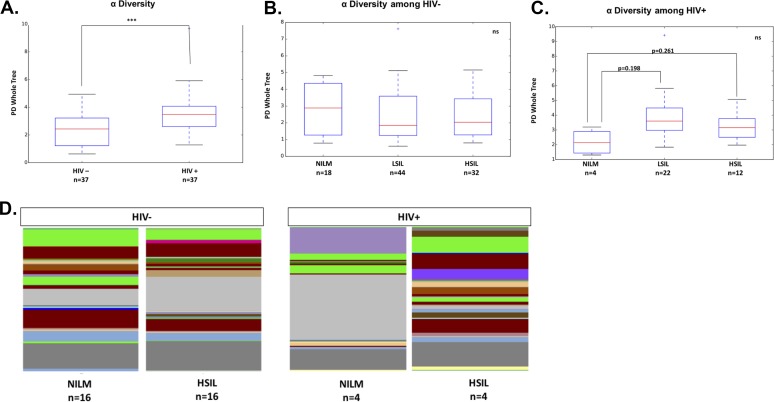
Alpha diversity measurements of cohort subgroups. (A) Relationship between HIV status and alpha diversity of cervical bacteria. (B) Relationship between cervical cytology and bacterial alpha diversity in HIV− individuals. (C) Relative abundance of genus-level reads differentiated by cervical cytology in HIV+ and HIV− individuals. Statistical significance is indicated as follows: ns, not significant; *, *P* < 0.1; **, *P* < 0.05; ***, *P* < 0.01. (D) Relative bacterial diversity of cervical microbiota graphed as a function of HIV status. Each color represents a different taxonomic family as defined by deep sequencing of the 16S gene.

Alpha diversity metrics, Chao1, observed OTUs, and PD Whole Tree, displayed higher (*P* = 0.009) bacterial richness in HIV+ patients than in HIV− patients (Fig. 4). A subset of these samples was matched such that the HIV− and HIV+ groups consisted of the same number of samples, with the same average age, and the same contribution of each cervical cytology to help to control for effects of these confounding variables and to ensure that differences in diversity estimates are not due to differences in sample size. In this matched subset, estimates also displayed higher (*P* = 0.003) bacterial richness in HIV+ patients.

Alpha diversity metrics were similar (*P* > 0.50) for the samples from patients at different cervical cytology grades (NILM, LSIL, or HSIL) in both matched and unmatched sets. When alpha diversity metrics were compared between cervical cytology groups separately for HIV+ samples, LSIL and HSIL trended toward a higher diversity compared to NILM (*P* = 0.198 and *P* = 0.261, respectively). Analysis of age-matched, HIV+ NILM/HSIL pairs maintained this trend (*P* = 0.264; Chao1 *P* = 0.13). Comparison of the relative abundance of genus-level reads between these groups showed a noticeably more diverse profile for HSIL samples, which lack the dominance of *Lactobacillus* and *Haemophilus* seen in NILM samples.

**FIG 5:**
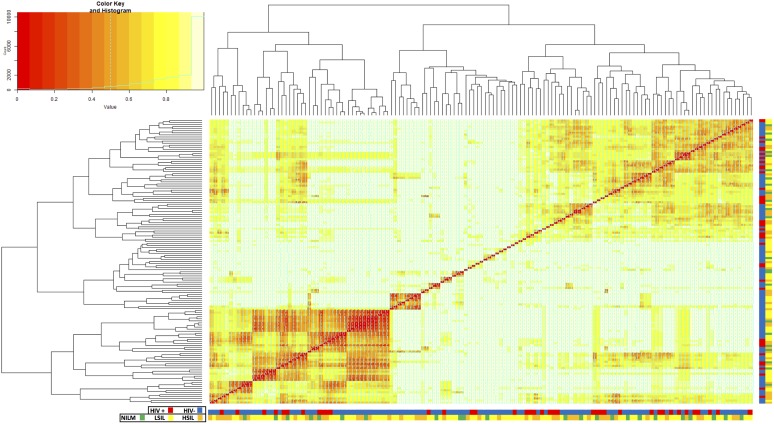
Heatmap of the Bray-Curtis distances between each sample (beta diversity). Samples are grouped into a similarity tree based on the abundance of each OTU. Lower values (red) indicate more similarity. HIV status and cervical cytology of each sample are indicated by color beneath each column and beside each row (HIV+ [red], HIV− [blue], NILM [green], LSIL [yellow], HSIL [orange]).

Beta diversity analysis showed that bacterial communities were quite varied between samples (Fig. 5); no discrete communities characterized a large number of samples. On average, the cervical bacterial communities of HIV-positive patients were shown to be significantly different from the communities of HIV-negative patients (*P* = 0.001). Similarly, patients who tested positive for HPV tended to have different bacterial communities from those who tested negative for HPV (*P* = 0.008). Bacterial communities were also shown to differ significantly depending on cervical cytology among HIV-positive patients (*P* = 0.05).

### Bacteria associated with cervical cytology states and/or HIV status.

**FIG 6:**
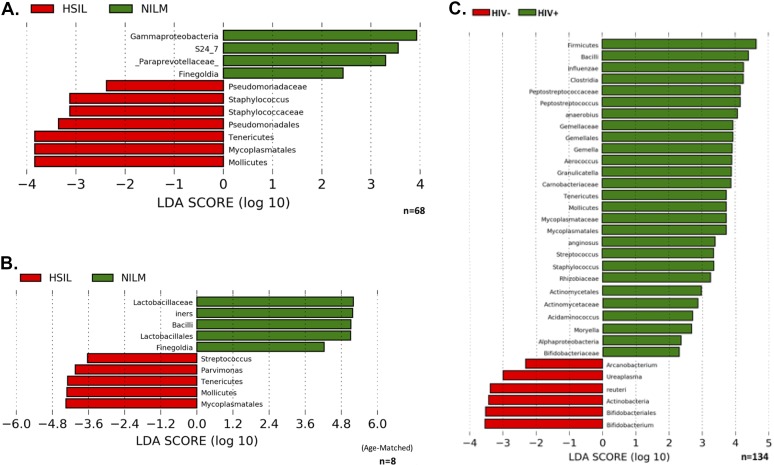
LEfSe linear discriminant analysis (LDA) scores. Microbes associated with cervical cytology status and/or HIV status are displayed. (A) Taxonomies differentiating bacterial microbiota in cytologically normal versus HSIL cervices. (B) Taxonomies differentiating bacterial microbiota in cytologically normal versus HSIL cervices in age-matched HIV+ patients. (C) Taxonomies differentiating bacterial microbiota in HIV− versus HIV+ cervices.

Linear discriminant analysis effect size (LEfSe) was used to identify bacterial taxonomies which differentiate cervical microbiota in normal individuals (NILM) from microbiota in patients with precancerous lesions (HSIL). The sum of reads at each taxonomic rank was considered. *Gammaproteobacteria*, s24_7, *Paraprevotellaceae* (nonverified taxonomy), and *Finegoldia* associated with NILM cervices, while *Pseudomoriadaceae*, *Staphylococcus*, and *Mycoplasmatales* associated with precancerous lesions. *Mycoplasmatales* were dominant among *Tenericutes*, resulting in the significant association seen between the phylum and cervical lesions. A distance-based redundancy analysis (db-RDA) analysis of bacterial communities as a function of HIV and/or cervical cytology is summarized in Fig. S2. LEfSe was then used to compare HIV+, age-matched pairs of NILM and HSIL patients to determine which bacteria may influence the development of lesions in high-risk, HIV+ populations. *Mycoplasmatales* were most strongly associated with cervical lesions in HIV+ patients, followed by *Parvimonas* and *Streptococcus*. In NILM patients, an abundance of *Lactobacillus*, especially Lactobacillus iners was found, and somewhat less significantly *Finegoldia*. LEfSe analysis of samples by HIV found several bacteria to be associated with being HIV+ (Fig. 6C). An abundance of non-*Lactobacillus* bacilli was the most significant differentiating taxonomy between HIV-positive and -negative samples. *Mycoplasma* was also associated with HIV+ individuals, supporting the significant difference in relative abundance between HIV-positive and -negative groups shown previously using a direct Kruskal-Wallis comparison. Interestingly, *Ureaplasma* (a member of *Mycoplasmatales*) and Lactobacillus reuteri were associated with HIV− patients, while other members of their respective families were associated with HIV+ patients. This suggests the existence of metabolic niches in the cervical microbiome which may be populated by pathogenic or nonpathogenic bacteria.

10.1128/mBio.02785-18.2 FIG S2 The bacterial community composition differences were analyzed in reference to cervical cytology and HIV status using db-RDA with the unweighted UniFrac distance matrix. (S2A) Db-RDA analysis of the bacterial community as a function of HIV status. (S2B) Db-RDA analysis of the bacterial community as a function of HIV status. (S2C) Db-RDA analysis of the bacterial community as a function of cytology (NILM versus LSIL or HSIL) at *P* = 0.849. (S2D) Db-RDA analysis of the bacterial community as a function of cytology (NILM versus LSIL or HSIL) at *P* = 0.05. Download FIG S2, TIF file, 1.1 MB. Copyright © 2019 Klein et al. 2019 Klein et al. This content is distributed under the terms of the Creative Commons Attribution 4.0 International license.

## DISCUSSION

We found that HPV was in high abundance in the cohort (Fig. 1A). All of the patients who were HIV positive were positive for one or more HPVs (Fig. 1A). Among the HIV− samples, HPV was associated with at least a 10-fold increase in *Bacteriodetes* and fusobacteria as well as a decrease in *Actinobacteria* (Fig. 1B and C). Previous studies support the conclusion that HPV affects the microbiome (24).

Certain members of the cervicovaginal microbiome are known to protect against infection and pathogenesis. The primary defense mechanisms of the cervicovaginal mucosa are antimicrobial peptides, a pH of less than 4.5, and a microbiome dominated by lactobacilli. An imbalance in these defenses can result in physiochemical changes that produce alterations of the vaginal mucosa and cervical epithelium (27). In particular, an abundance of Lactobacillus crispatus shows an inverse relationship with detectable or symptomatic HIV, HPV, or herpesvirus infection (25). This suggests that other cervicovaginal microbes may be important in preventing or enhancing the acquisition and pathogenesis of such infections. Microbes that are associated with enhanced pathogenesis have largely gone unidentified or unstudied, especially in the population most at risk, HIV-positive women in sub-Saharan Africa.

In this study, HIV was shown to have a significant effect on the cervical microbiome, increasing bacterial richness and decreasing beta diversity. These results are similar to what has been reported for the cervicovaginal microbiome and suggest that changes in the cervical epithelium microenvironment brought on by HIV exert some selective pressure on cervical bacterial communities (24–28). *Mycoplasma* was significantly more abundant in HIV-positive patients and was found to be one of the main categories of bacteria that differentiate the cervical microbiota of HIV-positive and HIV-negative individuals (Fig. 3). Interestingly, bacteria of the order *Bacilli*, of which *Lactobacillus* is a member, were strongly associated with HIV-positive patients. The absence of *Bacilli* reads classified as *Lactobacillus* among the significant factors of HIV-positive cervical microbiota suggests that this may be due to a shift from protective to nonprotective *Bacilli* in HIV+ individuals. When the cohort was analyzed without taking HIV status into account, cervical cytology did not appear to have a statistically significant association with differences in the cervical microbiome (Fig. 4B). However, when HIV was controlled for by separating analysis by groups of HIV-positive or HIV-negative patients only, differences in cervical bacterial communities that varied on the basis of cervical lesion status began to reach statistical significance (Fig. 4C). This suggests that development of precancerous cervical lesions is associated with a certain microbiota. Among these microbiota, *Mycoplasmatales* stood out as the most significant differentiator between the cervical microbiota of a cervix with precancerous lesions from a cervix without precancerous lesions (Fig. 6). Bacteria belonging to the order *Mycoplasmatales* also showed the clearest linear increase in abundance with development of more severe lesions in both HIV-positive and HIV-negative populations. The most common *Mycoplasmatales* to infect the urogenital tract of women are Mycoplasma genitalium and Mycoplasma hominis. M. genitalium and M. hominis are noncommensal bacteria commonly associated with cervicitis, BV, PID, and HIV infection, though M. genitalium has been much better studied (29–31). It is not well understood whether HIV promotes mycoplasma infection or persistence of an otherwise transient infection in an HIV-negative individual. One study found that HIV-positive women cleared M. genitalium infections more slowly than HIV-negative women did, and the infection recurred in 39% of the patients after clearance (32). The role of M. genitalium infection in influencing initial infection of HIV also remains unclear; however, a strong association between the severity of M. genitalium infection and HIV shedding from the cervix has been shown (33). What is clear is that M. genitalium infects the epithelia, disrupting tight junctions, and inducing a chronic inflammatory response. The potential for M. genitalium to influence replication of HIV suggests that host innate responses to M. genitalium infection may influence pathogenesis of other sexually transmitted infections. Induction of HPV in this way is particularly interesting based on the association between *Mycoplasma* and cervical lesions. Infection with M. genitalium increases the rate of infection with an HPV genotype associated with a high risk of developing cervical cancer (34). Recent work has shown that mycoplasma also increases the risk of development of cervical lesions, supporting the association we report in this study (34). *Mycoplasma* can establish persistent, intracellular infections in epithelial cells, which may lead to bacterial vaginosis and/or cervicitis. M. genitalium has been established as an independent, causal microbe responsible for cervicitis (35). This suggests that *Mycoplasma* may act as both an intracellular and extracellular stressor, particularly if coinfection with HPV has taken place. This interaction would most likely involve inflammatory cytokines induced by *Mycoplasma* infection. Further study is needed to determine whether the inflammatory cytokines induced by *Mycoplasma* infection include cytokines that are associated with precancerous cervical lesions.

*Mycoplasma* is a low-abundance microbe that has been shown to cause cervicitis. However, the lack of significant associations in previous metagenomic studies is largely due to a lack of optimization of statistical analyses for the presence of low-abundance microbes. In our study, *Mycoplasma* was a prominent result, likely due to the large HIV-positive proportion of the cohort, wherein immunosuppression allowed higher abundance of the bacteria to accumulate. There was a linear increase in the abundance of *Mycoplasmatales* from NILM to HSIL seen in both HIV-positive and -negative groups.

In this study, we took great effort to control for variation in the cervical microbiome so as to reduce confounding effects that might obscure the bacterial communities that were associated with HPV pathogenesis. The HIV-positive population is of particular interest, since they appear to show changed cervical microbiota associated with HPV pathogenesis (Fig. 2, 3, 4, and 6). Future studies, recruiting a cohort of all HIV-positive women with and without cervical lesions would be desirable in order to better characterize HIV-associated microbiota which promote HPV infection and progression to cervical cancer. Currently, few cervical microbiome cohort studies have been conducted in HIV-positive populations. It is clear that variables such as diet, genetic background, antibiotics or ART, can dramatically affect the microbiota and thus should be carefully controlled at the point of recruitment to the study.

Longitudinal studies of the cervical microbiome are needed to understand how microbe populations change over time, particularly in individuals with HSIL. Long-term longitudinal studies will allow determination of early changes in the cervical microbiota that may help predict the development of precancerous lesions. Because progression of HPV infection to cervical cancer is a process that takes decades, and in many individuals never reaches cancer at all, such a study would need to be large. Studies of the cervical microbiome can be further improved using metagenomic sequencing, rather than 16S or other targeted sequencing techniques that lack depth. 16S amplification ignores microbes that lack a gene to match the primers, for example, viruses, archaea, and eukaryotes are not accounted for. Because only a portion of one gene is being sequenced, the microbes present may be estimated only to the genus level or to a higher taxonomic level. Since the majority of medium- or large-scale cervicovaginal microbiome studies have used this method, the role of nonbacterial components of cervicovaginal microbiome in HPV infection and disease has not been characterized.

As the world’s HIV-positive population grows, cervical cancer is expected to become an even more significant problem, despite increasing coverage of antiretroviral treatment (ART). Compared to the risk reduction after ART seen in other AIDS-defining cancers like Kaposi’s sarcoma and non-Hodgkin’s lymphoma, the risk of cervical cancer is not significantly affected, and recurrence rates remain high with or without treatment (36–39). Understanding microbes that influence this environment will help identify cervical microbiota associated with low- and high-grade cervical lesions. This may allow certain cervical microbiota to be used as diagnostic markers for those at high risk of developing cervical cancer and for the development of preventative probiotic or antibiotic treatments that could control the cervical microbiome by promoting bacterial colonization with a microbiota associated with healthy cervical cytology. Our studies have identified a unique microbiota associated with HSIL. Data derived from our precise sampling of cervical lesions lead us to propose that *Mycoplasma* contributes to a cervical microbiome status that promotes HPV-related cervical lesions. These results suggest a greater influence of the bacterial microbiota on the outcome of HPV infection than previously thought.

## MATERIALS AND METHODS

### Participants and ethical precautions.

This study reports findings derived from a larger cross-sectional cohort study analyzing demographics of HPV and cervical cancer in HIV-positive and -negative women from rural and urban Tanzania.

The cervical microbiome study participants were part of a larger ongoing study to follow HIV- and HPV-associated cervical dysplasia in women at Ocean Road Cancer Institute (ORCI), the only cancer treatment hospital in Tanzania. Between March 2015 and February 2016, female patients undergoing cervical cancer screening were approached for enrollment in the study. Those who were pregnant, menstruating, under 18, reported being sick in the past 30 days, or had a preexisting, non-HIV, immunologic defect were excluded from the study. Disease histories as well as physical examinations were carried out to rule out any clinical symptoms or visible signs for these conditions. Samples were collected at three sites in Tanzania: ORCI in Dar es Salaam and rural clinics in Chalinze and Bagamoyo. A total of 144 cervical cytobrush samples obtained from these women were sequenced, of which 134 samples produced at least 1,000 reads and complete demographic data was available for the women. Of these, 132 had complete HIV data and cervical cytology reads.

### Demographic data collection.

All study participants gave informed consent and were evaluated by study clinicians. A set of pretested, standardized questionnaires was used to gather demographic data. All personal identifiers were removed from samples to ensure patient confidentiality. With the permission of the patients, medical history was retrospectively retrieved from hospital medical records. More than 30 variables were identified and assessed in the questionnaire. The current study uses only data collected regarding age and laboratory test results (pap smears, visual inspection with acetic acid [VIA], CD4 count, genotyping of HPV, results of serological testing for HIV-1).

### Specimen collection, HIV, CD4, and pap tests.

Blood samples were collected via venipuncture into acid-citrate-dextrose tubes and processed using centrifugation at the on-site study laboratory within 6 h of being drawn. The separated plasma was tested at the ORCI, as part of standard of care, using Standard Diagnostics HIV-1/2 3.0 detection kit and BD products CD4 FITC, CD8 PE, and CD3 Per CP antibodies to test the CD4 counts using a BD Accuri C6 Plus. Cervical cytobrush samples and pap smears were collected from all patients. Pap smears were examined by at least three trained cytologists and classified according to the pap classification protocol: negative for intraepithelial lesion or malignancy (NILM); atypical squamous cells of undetermined significance (ASC-US); low-grade squamous intraepithelial lesions (LSIL); atypical squamous cells but cannot exclude high-grade lesions (ASC-H); high-grade squamous intraepithelial lesions (HSIL). Cervical cytobrush specimens were placed in lysis buffer and then shipped to the Nebraska Center for Virology at the University of Nebraska-Lincoln (UNL) for processing.

### DNA isolation, 16S rRNA library preparation, and sequencing of the V4 region.

Cervical cytobrush samples were vortexed and separated from the brush with lysis buffer. DNA was extracted from the lysis buffer using the Qiagen Tissue extraction kit (Dneasy) according to the manufacturer’s protocol. The DNA concentration was determined by UV spectrophotometer at 260/280 nm.

DNA was then used for tag sequencing of the V4 hypervariable region of the 16S rRNA gene. A 250-bp section of the V4 region was amplified using universal primers described in reference 40. The PCRs were performed in 25 μl. The cycling conditions were as follows: an initial denaturation of 98°C for 3 min, followed by 25 cycles, with 1 cycle consisting of denaturation at 98°C for 30 s, annealing at 55°C for 30 s, and extension at 68°C for 45 s, and then a final elongation of 68°C for 4 min. Following amplification, PCR products were analyzed on a 2% agarose gel to confirm correct product size. Normalized amplicons (1 to 2 ng/μl) from 144 samples were pooled together using an epMotion M5073 liquid handler (Eppendorf AG, Hamburg, Germany). Pooled libraries were sequenced using the Illumina MiSeq platform using the dual-index sequencing strategy outlined by Kozich et al. (40).

### HPV genotyping.

To determine HPV status, DNA samples were subjected to HPV redundant primer using the GP5+/GP6+ primer set, which detect up to 40 different mucosal HPVs (41–43). Samples found to be HPV positive were genotyped for HR-HPVs (types 16, 18, 30, 31, 33, 35, 39, 45, 51, 52, 56, 58, 59, and 66) and LR-HPVs (types 6 and 11) using a low-cost multiplex PCR assay (44).

### Data processing and bacterial community analysis.

The sequencing data obtained from the sequencer was subsequently analyzed using the Illumina MiSeq data analysis pipeline developed by the Fernando lab (described in detail at https://github.com/FernandoLab). Briefly, initial quality filtering was carried out to remove sequences that had ambiguous bases, incorrect lengths, and inaccurate assemblies. Subsequently, the quality-filtered reads were run through the UPARSE pipeline (http://www.drive5.com/uparse/) and subjected to chimera filtering and OTU clustering (at a similarity threshold of 97%), followed by the generation of an OTU table. Taxonomy was assigned to the OTUs using the assign_taxonomy.py command available in QIIME using the latest version of the Greengenes database (May 2013).

### Statistical analyses.

The OTU table was rarefied across samples to the lowest sample depth (1,000 reads) using QIIME based on the Mersenne Twister pseudorandom number generator. All statistical analyses were performed with samples at an even depth. Bar charts summarizing average taxonomic makeup of samples by HIV status and cervical cytology were constructed from the rarefied OTU table in QIIME. Heatmaps showing the relative abundance of bacterial taxonomic families were constructed using the “plot_ts_heatmap” command using the mctoolsR package for R. Differences in bacterial families by HIV status or cervical cytology were evaluated using the “taxa_summary_by_sample_type” command in mctoolsR using Kruskal-Wallis. Families with less than 1% abundance were excluded in this analysis. Alpha diversity estimators Chao1, observed OTUs, and PD whole tree and rarefaction curves were calculated for the overall bacterial community using QIIME. Good’s coverage test was performed to evaluate whether adequate sampling depth was achieved. Mean alpha diversity estimates for HIV-positive, HIV-negative, NILM, LSIL, and HSIL groups were compared using nonparametric two-sample *t* tests using Monte Carlo permutations in QIIME. The weighted and unweighted UniFrac distance matrix for bacterial communities were calculated using QIIME. Even depth across samples avoided biases that could be encountered when using the UniFrac metric (45). Bacterial community composition differences were evaluated using the unweighted UniFrac distance matrix as an input for a distance-based redundancy analysis (db-RDA) in QIIME, where HIV status, cervical cytology, and HPV status were used as main effects. A heatmap was generated using the heatmap.2 command in the “ggplots” package for “R” using the Bray-Curtis distance matrix to visualize relationships between samples. Significance was declared at *P* ≤ 0.1 throughout this study. The linear discriminant analysis effect size (LEfSe) was used to identify specific OTUs that differed HIV status and cervical cytology (46). LEfSe uses a nonparametric factorial Kruskal-Wallis rank sum test followed by a linear discriminant analysis to identify both statistically significant and biologically relevant features. The relative abundances of the OTUs were used as input for LEfSe. Demographic data were examined using odds ratio and an associated *P* value to test for factors associated with HIV status and/or a positive VIA status. All *P* values are reported as FDR-corrected *P* values.

### Ethics statement.

All human subject protocols were approved by safety committees at the Ocean Road Cancer Institute (ORCI) and UNL in accordance with the Helsinki Declaration. Participation by patients was entirely voluntary, and written patient consent was required for inclusion in the study.
