# Supplementary figures and images for: Relationship between the Cervical Microbiome, HIV Status, and Precancerous Lesions
Source: mBio. 2019 Feb 19;10(1):e02785-18. doi: 10.1128/mBio.02785-18 (PMC6381280; doi:10.1128/mBio.02785-18)

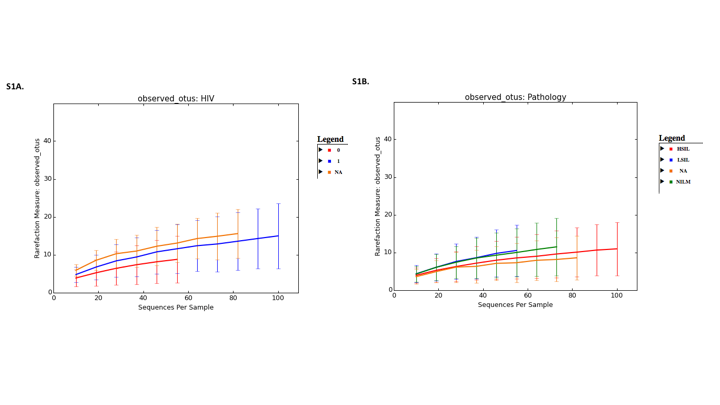

Supplement: FIG S1 [file mBio.02785-18-sf001.tif]

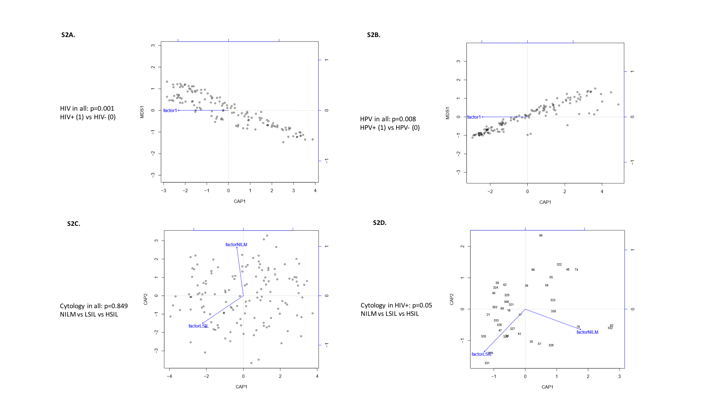

Supplement: FIG S2 [file mBio.02785-18-sf002.tif]
